# Evaluation of immune cell subsets of cancer patients treated with Avelumab, a fully human IgG1 anti-PD-L1 MAb capable of mediating ADCC of human tumor cells

**DOI:** 10.1186/2051-1426-3-S2-P254

**Published:** 2015-11-04

**Authors:** Lauren Lepone, Renee Donahue, Italia Grenga, Caroline Jochems, Kwong-Yok Tsang, Simon Metenou, Jacob Richards, Christopher R Heery, Ravi Madan, James L Gulley, Jeffrey Schlom

**Affiliations:** 1 Laboratory of Tumor Immunology and Biology, Center for Cancer Research, National Cancer Institute, National Institutes of Health, Bethesda, MD, USA; 2 National Cancer Institute, National Institutes of Health, Bethesda, MD, USA; 3 Genitourinary Malignancies Branch, Center for Cancer Research, National Cancer Institute, National Institutes of Health, Bethesda, MD, USA; 4 Genitourinary Malignancies Branch, National Cancer Institute, National Institutes of Health, Bethesda, MD, USA

## Background

Several monoclonal antibodies (MAbs) with demonstrated clinical anti-cancer activities have been engineered as fully human IgG1 entities to also encompass their potential to mediate antibody-dependent cell-mediated cytotoxicity (ADCC) of human tumor cells. Avelumab (MSB0010718C, EMD Serono, Pfizer) is a fully human IgG1 MAb targeting the co-regulatory protein Programmed Death-Ligand 1 (PD-L1), and is thus distinct from other MAbs targeting the PD-L1/PD-1 axis currently being evaluated in clinical trials. Concern has been raised that an anti-PD-L1 antibody capable of inducing ADCC may negatively affect PD-L1 expressing immune cell subtypes. This work is intended to determine if there is any validity to this concern.

## Methods

The clinical activity of Avelumab, observed in several tumor types in ongoing clinical studies such as NCT01772004, has been and will be reported elsewhere. In the studies reported here, Avelumab is shown to mediate ADCC of several types of human tumor cell lines (e.g., breast, lung, bladder carcinomas) in vitro, with tumor cell lysis mediated mainly by human CD16+ monocytes and natural killer (NK) cells. Since some human immune cell subsets express PD-L1 on their surface, studies were undertaken to evaluate changes in the frequency of immune cell subsets in peripheral blood mononuclear cells (PBMC) from cancer patients pre- vs post-treatment with Avelumab. Immune cells evaluated were PD-L1 positive and PD-L1 negative subsets of the following: CD4+ T cells, CD8+ T cells, NK cells, regulatory T cells (Tregs), myeloid-derived suppressor cells (MDSC), natural killer T cells (NKT), plasmacytoid dendritic cells (DC), conventional DC, and B cells.

## Results

Forty-two post-treatment PBMC samples were evaluated as follows: pre vs 1 dose of Avelumab (day 15, n = 19); pre vs 3 doses of Avelumab (day 43, n = 14); and pre vs 9 doses of Avelumab (day 127, n = 16). In all cases there were no statistical differences pre- vs post-treatment in any immune cell subset, and at any time point analyzed, regardless of whether the immune subset expressed PD-L1 or not. In addition, no changes were observed in absolute lymphocyte counts at any time point analyzed. Statistical analysis of all relevant immune cell subsets will be presented.

## Conclusion

While immune cell subsets pre- vs post-treatment continue to be analyzed in various patient cohorts, these studies provide evidence that Avelumab, a fully human IgG1 MAb, capable of mediating ADCC, can be administered safely to cancer patients without altering the balance of numerous PBMC immune cell subsets.

